# Life Essentials 8 score and risk of metabolic syndrome: A dose-response analysis in the US population

**DOI:** 10.1371/journal.pone.0312674

**Published:** 2024-10-31

**Authors:** Feng Jia, Jiaxuan Sun, Xiangliang Liu, Yahui Liu

**Affiliations:** The First Hospital of Jilin University, Changchun, China; PRISM CRO, PAKISTAN

## Abstract

**Background:**

There is limited research on the relationship between Life Essentials 8 (LE8) score and metabolic syndrome (MetS). Our aim is to examine the association between LE8 cardiovascular health metrics and risk of MetS in a nationally representative sample.

**Methods:**

We conducted a cross-sectional study using data from 23,253 adults aged ≥20 years from the National Health and Nutrition Examination Survey (2005–2018). LE8 score (range 0–100) was calculated based on the American Heart Association’s definitions of ideal cardiovascular health behaviors (physical activity, diet, smoking, and body mass index) and factors (total cholesterol, blood pressure, fasting plasma glucose, and fasting triglycerides). Metabolic syndrome comprises a cluster of metabolic disorders, including obesity, hypertension, hyperglycemia, and dyslipidemia. Multivariable logistic regression and restricted cubic spline models, mediation analysis, subgroup analysis and weighted quantile sum (WQS) regression were used to assess the relationship between LE8 score and MetS risk.

**Results:**

A total of 23,253 participants were included, of whom 7,932 had MetS and 15,321 did not. The average age of the participants was 50.7 years (standard deviation (SD) 12.3), with 49.24% being male. Participants with high LE8 category (80–100 points) had 98% lower odds of having MetS compared to those with low LE8 category (0–49 points) after adjusting for potential confounders (adjusted odds ratio [OR]: 0.02; 95% confidence interval [CI]: 0.02–0.03; P < 0.001). There was a monotonic decreasing dose-response relationship between LE8 score and predicted probability of MetS (P-overall <0.001; P-nonlinear <0.001). Several biomarkers including serum albumin, uric acid and neutrophil count emerged as potential mediators.

**Conclusions:**

While our studies suggest a potential association between cardiovascular health factors and reduced MetS risk, the cross-sectional nature of our study limits causal inferences. The LE8 score could still serve as a useful screening tool to identify individuals at high risk for MetS, facilitating targeted prevention and treatment strategies.

## Introduction

Metabolic syndrome (MetS) has become a major public health challenge worldwide. It is characterized by a cluster of interconnected physiological, biochemical, clinical, and metabolic factors that directly increase the risk of cardiovascular atherosclerotic diseases, type 2 diabetes mellitus, and all-cause mortality [1]. The underlying drivers of MetS are overweight/obesity, insulin resistance, hypertension, and dyslipidemia [2]. In the United States, approximately 35% of adults and 50% of those aged 60 years or older met the criteria for MetS [3]. Considering the high prevalence and deleterious consequences of MetS, early identification of at-risk individuals is of great significance.

The American Heart Association (AHA) proposed the concept of "Life Essentials 8" (LE8), which constitutes four health behaviors (diet, physical activity, nicotine exposure, and sleep) and four health factors (BMI, cholesterol, blood pressure, and fasting glucose) [4]. The LE8 score, ranging from 0 to 100, provides a composite measure of overall cardiovascular health (CVH). Previous studies have reported a strong association between better LE8 score and lower risk of cardiovascular disease (CVD) and mortality [5, 6]. Studies have shown that various components of cardiovascular health, such as a healthy diet, regular exercise, appropriate weight management, and blood pressure control, are closely associated with the risk of metabolic syndrome [7, 8]. However, while the LE8 score shows promise as a predictive tool for MetS, there is a lack of research examining its biological underpinnings. Therefore, further studies are needed to elucidate the biological mechanisms involved and to establish the theoretical basis for the predictive value of LE8 in MetS.

Therefore, in this study, we aimed to investigate the relationship between LE8 score and MetS using data from the continuous National Health and Nutrition Examination Survey (NHANES). We further explored the potential mediating roles of serum albumin, uric acid, and neutrophils in the hypothesized association. Quantile weighted survival regression was also conducted to assess the cumulative effects and weights of individual LE8 components on MetS. Findings from this study could help elucidate the predictive value of LE8 on MetS and its potential biological basis. The evidence may facilitate early prevention and treatment of MetS in clinical practice.

## Methods

### Study population

The data for this study were extracted from the publicly available NHANES database. Detailed information about the database’s study design and additional resources can be found on the official website at https://www.cdc.gov/nchs/nhanes/index.htm.. NHANES is a large cross-sectional survey conducted by the National Center for Health Statistics (NCHS) under the Centers for Disease Control and Prevention (CDC) in the United States. The survey follows a biennial cycle. This project is based on a complex multistage probability sampling design and aims to assess the nutritional and health status of the US population. All methods were performed in accordance with relevant guidelines and regulations.

**Fig 1:**
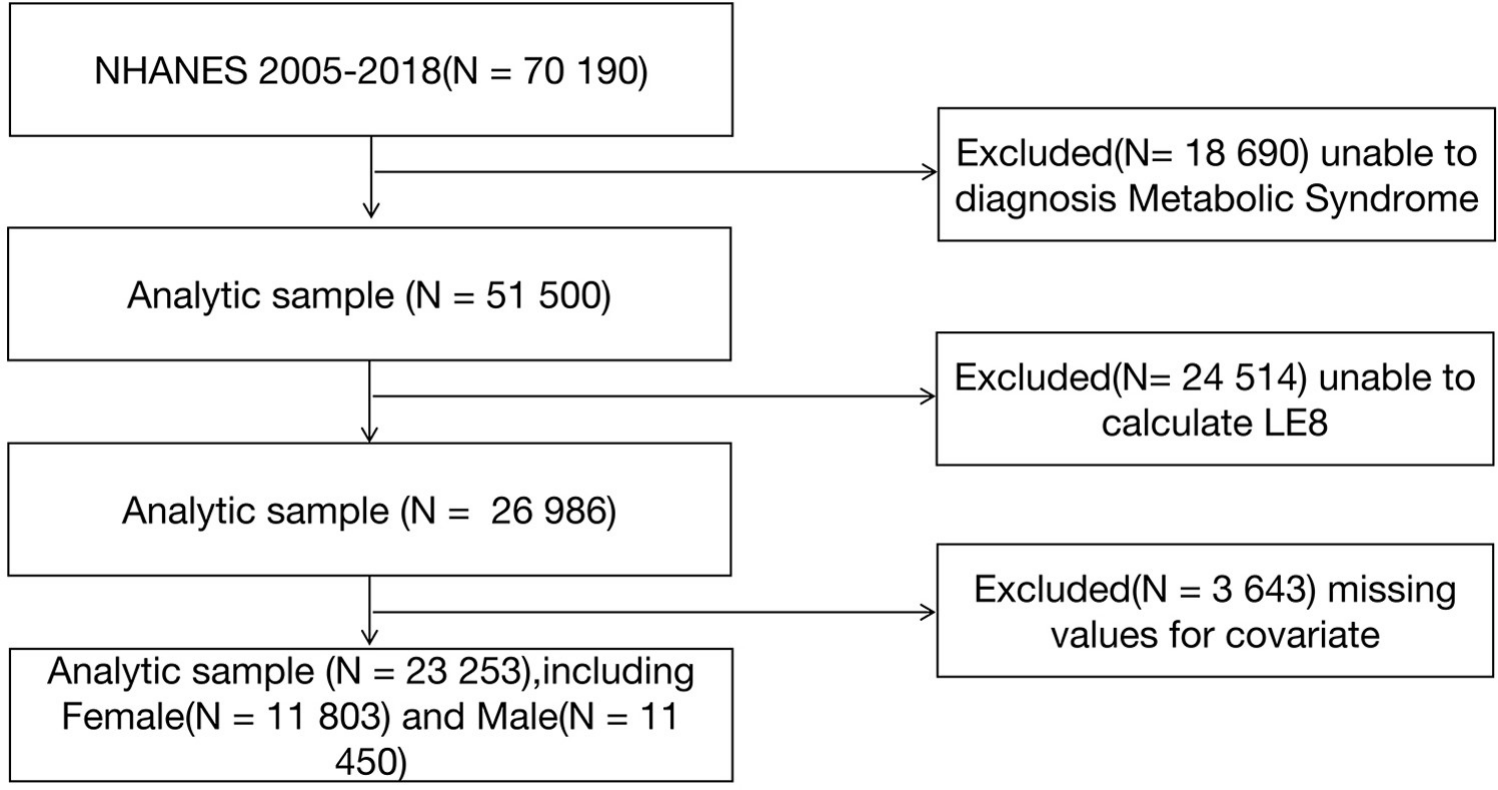
The flowchart of study and excluded participants, from NHANE 2005–2018.

Data from seven NHANES cycles (2005–2006, 2007–2008, 2009–2010, 2010–2012, 2013–2014, 2015–2016, 2017–2018) were collected for this study. A total of 70,190 individuals participated in the NHANES survey between 2005 and 2018. Among them, 18,690 participants were excluded from the analysis due to the absence of a metabolic syndrome diagnosis. Additionally, 24,514 participants were excluded from the analysis due to missing data that prevented the calculation of the LE8 Score. Furthermore, 3,643 participants were excluded from the analysis due to missing data on other covariates. Ultimately, a total of 23,253 participants were included in this study, comprising 11,450 males and 11,803 females. More details can be found in **Fig 1**.

### Measurement of LE8

The LE8 component consists of four health behaviors (nicotine exposure, sleep duration, physical activity, diet) and four health factors (non-high-density lipoprotein cholesterol, BMI, blood pressure, blood glucose). The detailed algorithm for the eight indicators of the LE8 score in the NHANES database has been published previously (**S1 Table**). Briefly, the scoring range for each CVH indicator is 0–100 points, and the final LE8 score is calculated as the arithmetic mean of the eight CVH indicators. A final score between 80 and 100 points is defined as a high-CVH group, a score between 50 and 79 points is defined as a moderate-CVH group, and a score between 0 and 49 points is defined as a low-CVH group [9]. This study uses the same cut-off points and definitions to further evaluate the health behaviors and factors in the LE8 components.

The dietary indicator is evaluated using the Healthy Eating Index-2015 (HEI-2015). The HEI-2015 is constructed using dietary intake data collected from two 24-hour dietary recall questionnaires and food equivalence data provided by the USDA. Using a previously published detailed algorithm, the HEI-2015 score was obtained [10]. For more details, see **S2 Table**. Information on nicotine exposure, sleep duration, physical activity, medication history, history of diabetes and other data are obtained from self-reported questionnaires. Physical activity is evaluated based on the duration and frequency of moderate or vigorous level activity in the past 30 days as reported in the questionnaire. Blood sugar, glycated hemoglobin levels, and lipids are obtained from blood tests sent to the central laboratory. Height, weight, blood pressure, etc. are obtained from medical examinations conducted on mobile examination vehicles.

### Metabolic syndrome (MetS) criteria

In accordance with prior guidelines, we utilized the diagnostic criteria for MetS as proposed by the American College of Endocrinology (ACE), specifically the National Cholesterol Education Program’s Adult Treatment Panel III (NCEP ATP III). This standard was established by the National Cholesterol Education Program in 2001 and was revised in 2005, whereby participants meeting three or more of the following criteria were diagnosed with MetS: 1). Hypertriglyceridemia: Serum triglycerides ≥150 mg/dL (1.7 mmol/L), or requiring treatment with lipid-lowering medication; 2). Central obesity: Waist circumference ≥102 cm for men and ≥88 cm for women; 3). Reduced high-density lipoprotein cholesterol (HDL-C): Serum HDL-C levels < 40 mg/dl (1.03 mmol/L) for men and < 50 mg/dl (1.29 mmol/L) for women; 4). Hypertension: Systolic blood pressure (SBP) ≥130 mmHg, diastolic blood pressure (DBP) ≥85 mmHg; 5). Hyperglycemia: Fasting plasma glucose ≥100 mg/dl (5.6 mmol/L), or requiring antidiabetic medication [11].

### Covariates

Neutrophil levels in blood samples were measured and recorded by professional technicians using an automatic hematology analyzer. The concentration of serum Albumin was determined using the dye-binding method. The concentration of uric acid in blood samples was measured using a urease-mediated oxidase method. More details on sample collection can be found at https://www.cdc.gov/nchs/data/nhanes/nhanes3/cdrom/nchs/manuals.. To further accurately evaluate the relationship between LE8 Score and MetS, the following confounding factors were adjusted: Gender (Male/Female), age ([20,40), [40,60), ≥60), race/ethnicity (Mexican American, Non-Hispanic Black, Non-Hispanic White, Other Hispanic, Other Race ‐ Including Multi-Racial), educational level (low high school, high school, college or above); poverty income ratio (PIR) (<1,1–3,≥3), alcohol consumption (Former drinker (individuals who used to drink but have stopped), Never drinker (individuals who have never consumed any alcoholic beverage), Mild drinker (daily alcohol consumption between 1 and 2 standard drink units), Moderate drinker (daily alcohol consumption not exceeding 4 standard drink units for men and 3 for women), Heavy drinker (daily alcohol consumption exceeding 4 standard drink units for men and 3 for women)).

### Statistical analysis

Following the complex probability stratified sampling design of the NHANES database, we conducted a weighted analysis on the eligible NHANES (2005–2018) data included in the final. Two-day dietary interview weights (WTDR2D), strata (SDMVSTRA), and primary sampling units (SDMVPSU) were considered to account for the complex design. The weight analysis involves the following steps: 1) Identifying the time range of the analysis variables: First, determine the NHANES survey cycles that encompass all analysis variables. Since our study combines data from multiple time periods, we need to ensure that all variables are recorded in each period. 2) Calculating combined weights: For data merged from multiple periods, we adjust and calculate sample weights according to NHANES guidelines. Specifically, we use the recommended calculation method, adjusting each period’s weight according to the length of its survey cycle. For example, if merging data from two 2-year cycles (e.g., 2001–2002 and 2003–2004), we divide each cycle’s weight by its respective length (i.e., by 2), then sum these adjusted weights to obtain the combined sample weight. 3) Applying sample weights, stratification variables, and primary sampling units: During statistical analysis, we apply the combined sample weights (WTDR2D), stratification variables (SDMVSTRA), and primary sampling units (SDMVPSU) to ensure the representativeness and accuracy of the analysis results.

Continuous variables are expressed as weighted means (±standard deviation), with statistical differences described using weighted t-tests. Categorical variables are shown as sample numbers (weighted percentages), with statistical differences described by Rao-Scott chi-squared test. Weighted univariate and multivariate logistic regression models were constructed to explore the relationship between LE8 Score, serum neutrophil levels, albumin levels, uric acid levels, and MetS. The univariate model did not adjust for any covariates; the multivariate model adjusted for gender, age, race/ethnicity, educational level, PIR and drinking level. To validate the association between albumin, uric acid, neutrophils, and MetS, we performed trend tests. Additionally, an RCS model was constructed to investigate the dose-response relationship between LE8 Score and MetS. Mediation analysis was employed to determine whether neutrophils, albumin, and uric acid levels mediated the relationship between LE8 Score and MetS. The indirect effect (IE) refers to the influence of neutrophils, albumin, and uric acid levels on MetS through the LE8 score. The direct effect (DE) represents the impact of LE8 on MetS after controlling for neutrophils, albumin, and uric acid levels. A significant IE indicates a significant mediating effect.

A WQS regression model was used, constructing a weighted index to estimate the mixed exposure effects related to all predictor factors of MetS. *β*_*o*_ represents the intercept, *β*_1_ represents the regression coefficient of the WQS model. g(μ) represents any monotonic differentiable link function and *φ*_*i*_ represents the weight of the i-th component. *φ*_*i*_ represents the quartiles of each LE8 component, (*φ*_*i*_ = 1,2,3,4) = 1, 2, 3, 4 represent the 1st, 2nd, 3rd or 4th quartile respectively. z′ and Φ represent the vector of covariates and the vector of regression coefficients respectively. c represents the number of LE8 indicators included in the analysis, then $\sum_{i=1}^{c}\bar{\omega_{i}}\varphi_{i}$ represents the sum of c weighted quantiles. We hypothesized a linear function fitting a Gaussian distribution, randomly assigned the data to a training set (60%) and a validation set (40%), and estimated the weights of the 8 LE8 components in the training set [12].


$$\text{g}(\mu )=\beta_{o}+\beta_{1}(\sum_{i=0}^{c}\omega_{i}\varphi_{i})+z\text{'}\Phi$$

$$WQS=\sum_{i=1}^{c}\bar{\omega_{i}}\varphi_{i}$$

To examine the relationship between LE8 Score and MetS across different populations, we conducted subgroup analyses and interaction tests. Age, sex, race/ethnicity, education level, and PIR were selected as primary stratification variables. Logistic regression models were employed to assess the relationship between LE8 Score and MetS for each subgroup. Interactions between LE8 Score and each stratification variable were incorporated into the models to test for differences in these relationships across subgroups. An interaction term with a p-value <0.05 was considered indicative of a significant interaction effect.

All statistical analyses and plots were conducted using R software (version 4.2.3.), all statistical tests were two-sided, and p-value<0.05 was considered statistically significant.

## Results

### Baseline characteristics

In this study, patients were categorized into two groups: those with MetS and those without MetS. Among the 23,253 participants, there were 11,450 males and 11,803 females. Compared with the group without MetS, participants in the MetS group were more likely to be male, over 60 years old, non-Hispanic white, have a high school education or above, have a PIR value above 3, and moderate drinkers. And compared to the non-MetS group, the MetS group had lower sleep duration score, lower BMI score and HEI-2015 score, lower levels of blood lipids score, blood glucose score, blood pressure score and physical activity score, and lower albumin levels, but higher serum neutrophil and albumin levels. More specific baseline details are available in **Table 1**.

**Table 1:** Survey-weighted baseline characteristic of the study population.

	Level	No Metabolic Syndrome	Metabolic Syndrome	P-value
Sex (%)				<0.001
	Female	10.75(0.40)	19.05(0.66)	
	Male	49.51(0.52)	47.55(0.86)	
Age (%)				<0.001
	20–40	46.50(0.86)	20.63(0.77)	
	40–60	34.98(0.68)	43.84(1.04)	
	≥ 60	18.53(0.57)	35.53(0.83)	
Race/ethnicity (%)				<0.001
	Mexican American	7.71(0.59)	8.67(0.83)	
	Non-Hispanic Black	10.59(0.69)	8.68(0.66)	
	Non-Hispanic White	69.03(1.26)	72.57(1.40)	
	Other Hispanic	5.06(0.44)	4.52(0.47)	
	Other Race ‐ Including Multi-Racial	7.61(0.43)	5.55(0.49)	
Education level (%)				<0.001
	Lower than high school	12.55(0.61)	16.25(0.75)	
	High school	20.81(0.62)	27.64(0.99)	
	Higher than high school	66.64(0.98)	56.10(1.23)	
Poverty income ratio (%)				<0.001
	<1	13.12(0.57)	13.25(0.66)	
	1–3	33.30(0.88)	39.06(0.94)	
	≥3	53.58(1.08)	47.69(1.31)	
Drinking status (%)				<0.001
	Former drinker	10.75(0.40)	19.05(0.66)	
	Heavy drinker	22.67(0.65)	17.70(0.72)	
	Mild drinker	37.25(0.76)	37.21(1.13)	
	Moderate drinker	19.52(0.55)	14.41(0.66)	
	Never drinker	9.81(0.58)	11.62(0.67)	
LE8 score				<0.001
	Low (0–49)	5.07(0.26)	24.28(0.75)	
	Moderate (50–79)	64.24(0.81)	72.62(0.75)	
	High (80–100)	30.69(0.85)	3.11(0.34)	
Tobacco/nicotine exposure score (mean (SD))		71.70(0.62)	70.95(0.67)	0.35
Sleep health score (mean (SD))		84.01(0.35)	82.24(0.44)	<0.001
Body mass index score (mean (SD))		70.91(0.43)	38.51(0.53)	<0.001
Blood lipids score (mean (SD))		69.97(0.43)	51.52(0.52)	<0.001
Blood glucose score (mean (SD))		93.00(0.21)	71.66(0.56)	<0.001
Blood pressure score (mean (SD))		77.19(0.36)	54.07(0.49)	<0.001
HEI-2015 diet score (mean (SD))		41.23(0.60)	37.10(0.61)	<0.001
Physical activity score (mean (SD))		76.29(0.50)	64.58(0.83)	<0.001
Albumin (mean (SD))		43.07(0.05)	42.00(0.06)	<0.001
Uric Acid (mean (SD))		5.21(0.02)	5.91(0.02)	<0.001
Neutrophils (mean (SD))		4.09(0.02)	4.63(0.03)	<0.001

### Association between weighted LE8 score and MetS

In the univariate weighted logistic regression model, compared to the group with low CVH, the risk of developing MetS in the moderate CVH group and high CVH group was reduced by 76% (OR 0.24, 95%CI 0.21–0.26, P<0.001) and 98% (OR 0.02, 95%CI 0.02–0.03, P<0.001), respectively. In the multivariate weighted logistic regression model, compared with the low CVH group, the risk of developing MetS in the moderate CVH group and the high CVH group was reduced by 75% (OR 0.25, 95%CI 0.22–0.28, P<0.001) and 98% (OR 0.02, 95%CI 0.02–0.03, P<0.001), respectively. More details can be seen in **Table 2**.

**Table 2:** Association between weighted Life Essentials 8 score and metabolic syndrome.

	Univariable model			Multivariable model		
	OR (95% CI)	P-value	P for trend	OR (95% CI)	P-value	P for trend
LE8 score			<0.001			<0.001
Low CVH (0–49)	Reference			Reference		
Moderate CVH (50–79)	0.24(0.21,0.26)	<0.001		0.25(0.22,0.28)	<0.001	
High CVH (80–100)	0.02(0.02,0.03)	<0.001		0.02(0.02,0.03)	<0.001	

Notes: LE8 score, Life Essentials 8; CVH, Cardiovascular health; OR, Odds ratio. Multivariable model: Adjusted for sex, race/ethnicity, education attainment, poverty income ratio and alcohol status.

### Association between weighted weighted LE8 score and serum albumin, uric acid, neutrophils

As shown in **Table 3**, in the multivariate weighted linear regression model, compared to the group with low CVH group, both the moderate CVH group (β0.61, 95% CI 0.46–0.77, P<0.001) and the high CVH group (β0.98, 95% CI 0.78–1.18, P<0.001) were significantly correlated with the serum albumin concentration. In the multivariate weighted linear regression model, compared to the group with low CVH group, both the moderate CVH group (β-0.21, 95% CI -0.29–0.14, P<0.001) and the high CVH group (β-0.39, 95% CI -0.48–0.29, P<0.001) were significantly correlated with the serum uric acid concentration. In the multivariate weighted linear regression model, compared to the group with low CVH group, both the moderate CVH group (β-0.59, 95% CI -0.68–0.50, P<0.001) and the high CVH group (β-1.00, 95% CI -1.11–0.90, P<0.001) were significantly correlated with the neutrophils level.

**Table 3:** Association between weighted weighted LE8 score and serum albumin, uric acid, neutrophils.

	Univariable model		Multivariable model	
	Standardized Coefficients β(95% CI)	P-value	Standardized Coefficients β(95% CI)	P-value
Albumin				
Low CVH (0–49)	Reference		Reference	
Moderate CVH (50–79)	1.32(1.13,1.50)	<0.001	0.61(0.46,0.77)	<0.001
High CVH (80–100)	2.28(2.06,2.51)	<0.001	0.98(0.78,1.18)	<0.001
Uric acid				
Low CVH (0–49)	Reference		Reference	
Moderate CVH (50–79)	-0.39(-0.47,-0.31)	<0.001	-0.21(-0.29,-0.14)	<0.001
High CVH (80–100)	-1.04(-1.13,-0.95)	<0.001	-0.39(-0.48,-0.29)	<0.001
Neutrophils				
Low CVH (0–49)	Reference		Reference	
Moderate CVH (50–79)	-0.72(-0.81,-0.63)	<0.001	-0.59(-0.68,-0.50)	<0.001
High CVH (80–100)	-1.26(-1.36,-1.17)	<0.001	-1.00(-1.11,-0.90)	<0.001

Notes: LE8 score, Life Essentials 8; CVH, Cardiovascular health; Multivariable model: Adjusted for sex, race/ethnicity, education attainment, poverty income ratio and alcohol status.

### Association between weighted serum albumin, uric acid, neutrophils and MetS

Serum albumin, uric acid, and neutrophil levels were classified based on quartiles: Q1 (< 25th percentile), Q2 (25th-50th percentile), Q3 (50th-75th percentile), Q4 (> 75th percentile). Univariate weighted logistic regression model revealed that compared to the Q1 group of serum albumin concentration, the risks of developing MetS decreased by 27% (OR 0.73, 95% CI 0.64–0.82, P < 0.001) in the Q2 group, 38% (OR 0.62, 95% CI 0.56–0.69, P < 0.001) in the Q3 group, and 62% (OR 0.38, 95% CI 0.34–0.43, P < 0.001) in the Q4 group. Multivariate weighted logistic regression model revealed that compared to the Q1 group of serum albumin concentration, the risks of developing MetS decreased by 27% (OR 0.73, 95% CI 0.64–0.82, P < 0.001) in the Q2 group, 34% (OR 0.66, 95% CI 0.59–0.74, P < 0.001) in the Q3 group, and 51% (OR 0.49, 95% CI 0.43–0.55, P < 0.001) in the Q4 group. Moreover, both univariate and multivariate weighted logistic regression models showed a significant positive correlation (p < 0.001) between higher levels of serum uric acid and neutrophils, and the risk of developing MetS. More specific details can be seen in **Table 4**.

**Table 4:** Association between weighted serum albumin, uric acid, neutrophils levels and metabolic syndrome.

	Univariable model			Multivariable model		
	OR(95% CI)	P-value	P for trend	OR (95% CI)	P-value	P for trend
Albumin			<0.001			<0.001
Q1	Reference			Reference		
Q2	0.73(0.64,0.82)	<0.001		0.73(0.64,0.82)	<0.001	
Q3	0.62(0.56,0.69)	<0.001		0.66(0.59,0.74)	<0.001	
Q4	0.38(0.34,0.43))	<0.001		0.49(0.43,0.55)	<0.001	
Uric Acid			<0.001			<0.001
Q1	Reference			Reference		
Q2	1.74(1.57,1.94)	<0.001		2.02(1.80,2.27)	<0.001	
Q3	2.50(2.26,2.77)	<0.001		3.30(2.92,3.73)	<0.001	
Q4	3.90(3.51,4.34)	<0.001		5.76(5.04,6.58)	<0.001	
Neutrophils			<0.001			<0.001
Q1	Reference			Reference		
Q2	1.59(1.43,1.76)	<0.001		2.02(1.80,2.27)	<0.001	
Q3	2.02(1.81,2.26)	<0.001		3.30(2.92,3.73)	<0.001	
Q4	2.71(2.48,2.97)	<0.001		5.76(5.04,6.58)	<0.001	

Notes: OR, Odds ratio. Multivariable model: Adjusted for sex, race/ethnicity, education attainment, poverty income ratio and alcohol status.

### Nonlinearity analysis using RCS

**Fig 2:**
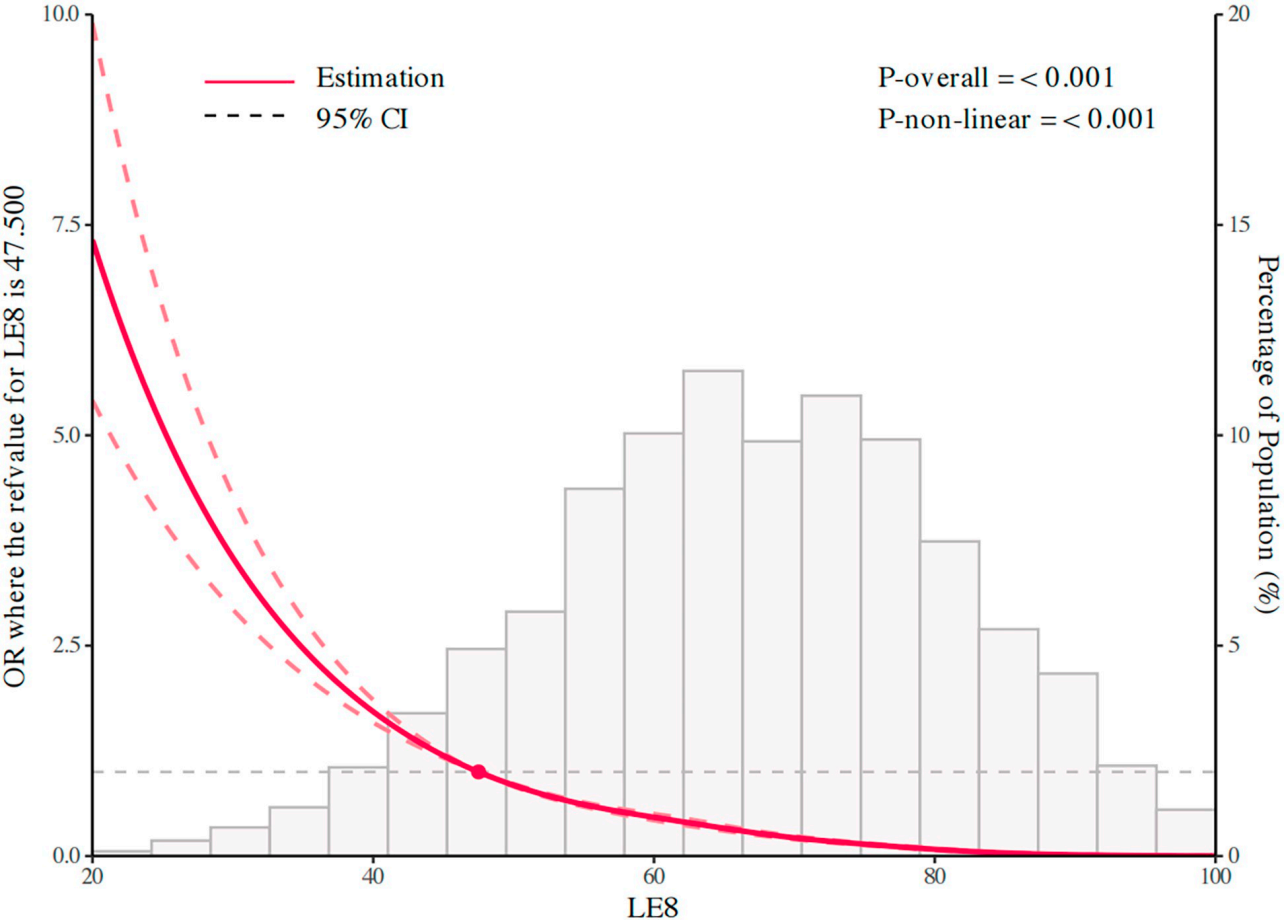
Association between Life Essentials 8 scores and metabolic syndrome using a restricted cubic spline regression model.

As shown in **Fig 2**, the RCS model was constructed to comprehensively evaluate the dose-response relationship between LE8 Score and MetS. The model established knots at the 5th, 35th, 65th, and 95th percentiles of LE8 Score, with the 5th percentile used as the reference value. The model was adjusted for gender, age, race/ethnicity, education attainment, PIR, and alcohol status. The results indicated a significant correlation and non-linear relationship between LE8 Score and MetS (p for overall association < 0.001, p for non-linear association < 0.001).

### Mediation analysis

**Fig 3:**
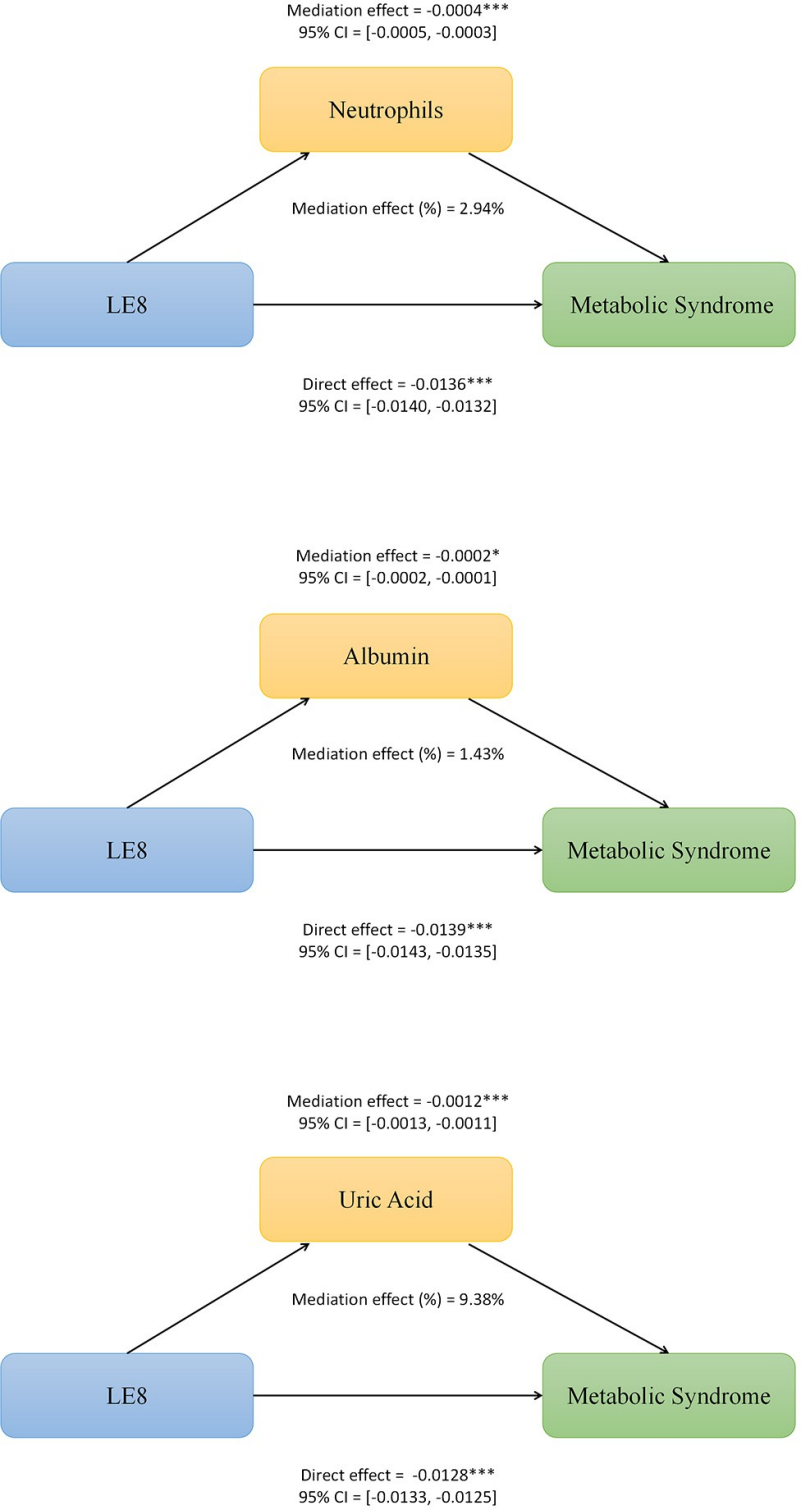
Path diagram of the mediation analysis models. A), serum albumin, B), uric acid, C), neutrophils levels partially mediates the relationship between Life Essentials 8 score and metabolic syndrome.

Mediation analysis was conducted to further investigate whether serum albumin, uric acid, and neutrophils mediated the relationship between LE8 Score and MetS. In this model, with LE8 Score as the independent variable and MetS as the dependent variable, serum albumin, uric acid, and neutrophils were considered as mediator variables. As shown in **Fig 3A**, serum albumin levels mediated the association between LE8 Score and MetS, explaining a total of 1.43% of the variance and demonstrating a significant mediating effect, with an indirect effect (IE) of -0.0002 (95%CI: -0.0002 to -0.0001). As shown in **Fig 3B**, serum uric acid levels mediated the association between LE8 Score and MetS, explaining a total of 9.38% of the variance and demonstrating a significant mediating effect, with an IE of -0.0012 (95%CI: -0.0013 to -0.0011). As shown in **Fig 3C**, serum neutrophil levels mediated the association between LE8 Score and MetS, explaining a total of 2.94% of the variance and demonstrating a significant mediating effect, with an IE of -0.0004 (95%CI: -0.0005 to -0.0003). More details can be seen in **Fig 3**.

### WQS regression

**Fig 4:**
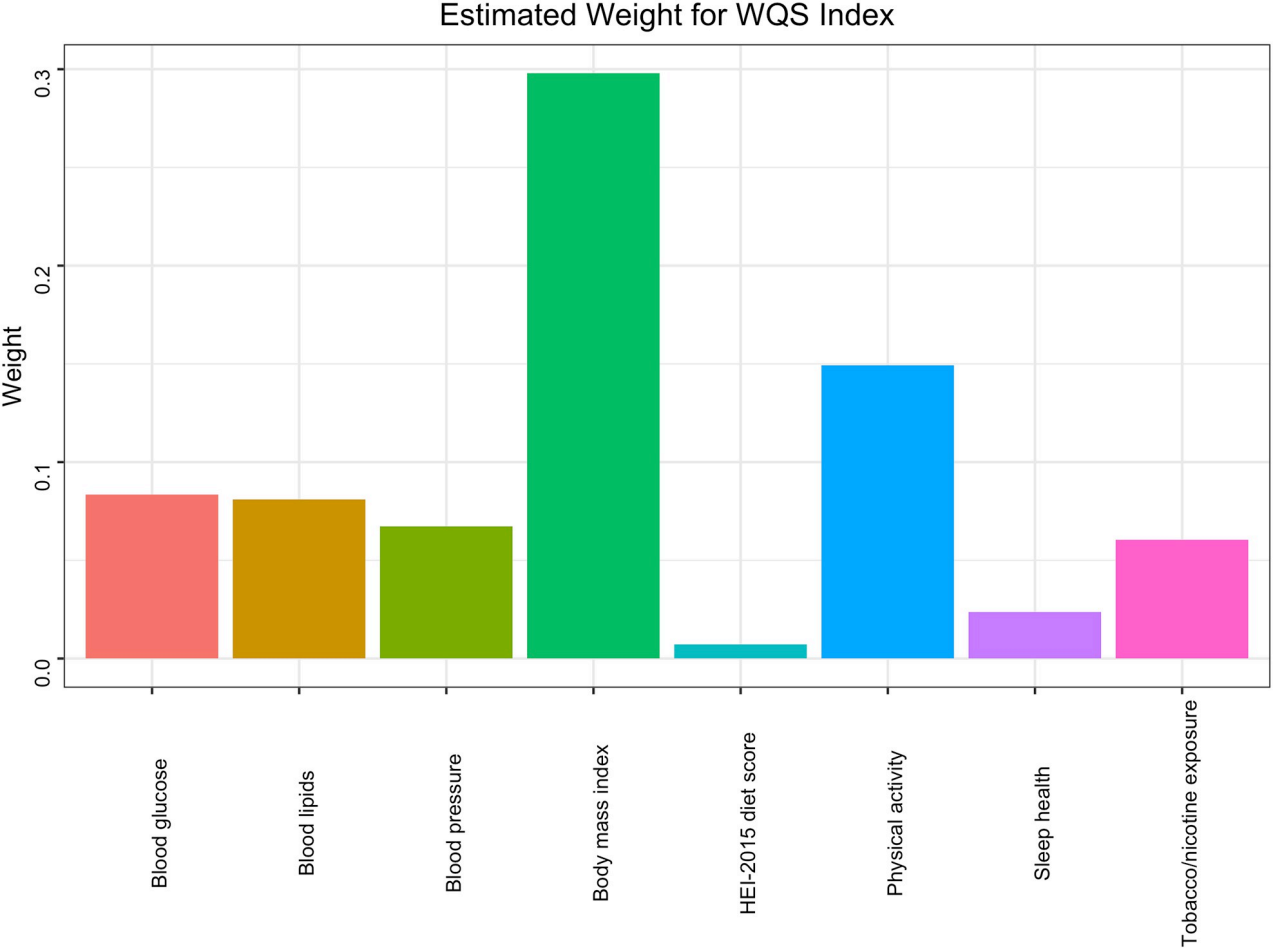
WQS model regression index weights for the metabolic syndrome, adjusted for sex, race/ethnicity, education level, poverty income ratio and alcohol status.

The WQS regression model was employed to construct a weighted index and assess the cumulative effects and weight contributions of different components in the LE8 Score on MetS. The variable adjustments in the model were consistent with the aforementioned multivariable model. The final results revealed that blood glucose (31.34%) and BMI (29.78%) had the highest weight contributions, while HEI-2015 (0.73%) had the lowest weight contribution. More specific details can be obtained in **Fig 4**.

### Subgroup analysis

**Fig 5:**
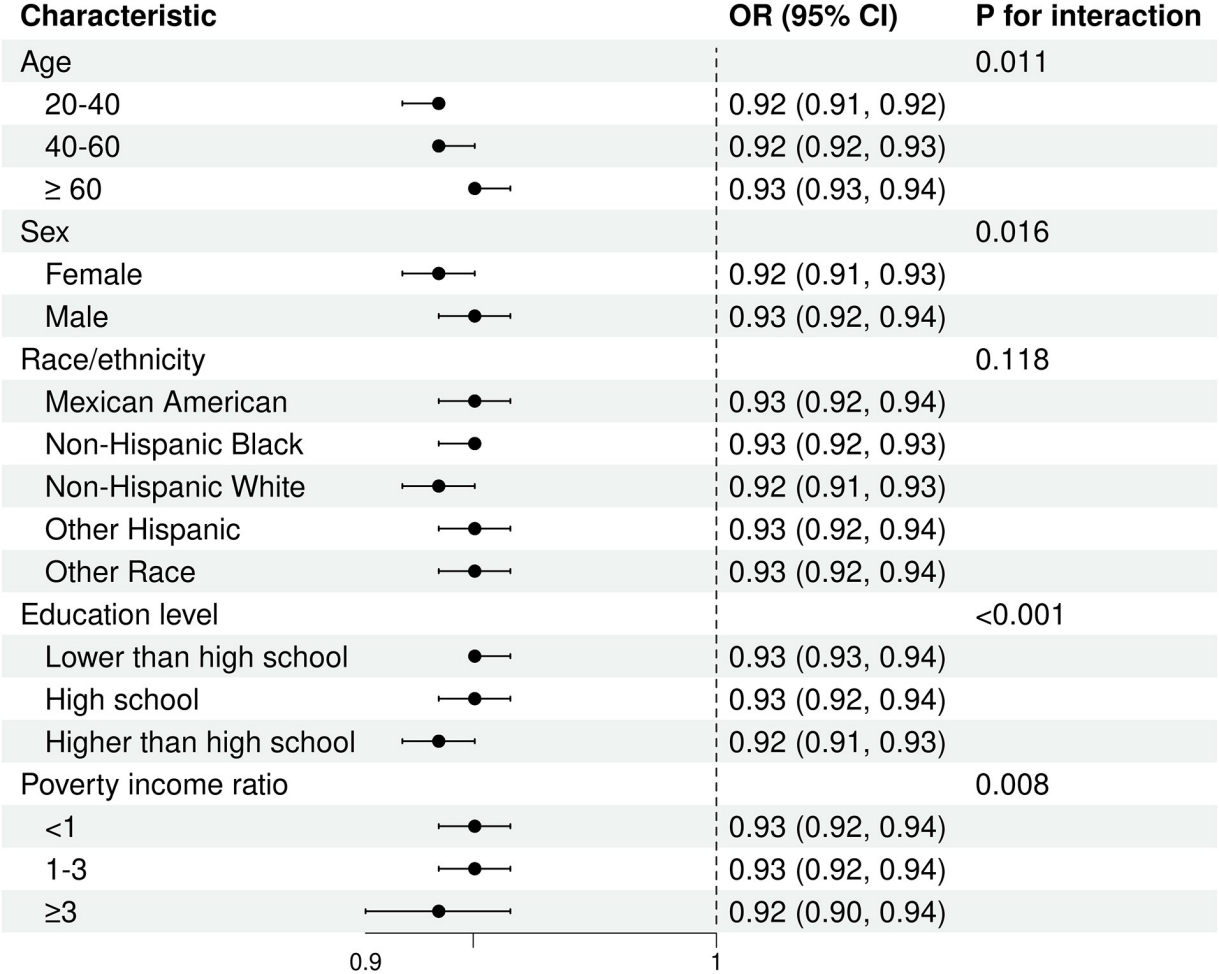
Subgroup analysis of the association of the Life Essentials 8 scores and metabolic syndrome. Each stratifcation was adjusted for age, sex, race/ethnicity, education level, and PIR.

Subgroup analysis were conducted based on age, sex, race/ethnicity, education level, and PIR. The results indicated a negative correlation trend between LE8 Score and MetS across all subgroups. Moreover, interaction analysis demonstrated significant interactions between LE8 Score and age, sex, education level, and PIR (P for interaction < 0.05). Further details can be found in **Fig 5**.

## Discussion

In this large nationally representative sample of 23,253 US adults from the continuous NHANES surveys, we found a robust inverse association between LE8 score and risk of MetS after adjusting for sociodemographic and behavioral confounders. Participants with LE8 scores in the high range (80–100 points) had 98% lower odds of having MetS compared to those with scores in the low range (0–49 points) (adjusted OR: 0.02; 95% CI: 0.02–0.03; P < 0.001). Using RCS models, we characterized a monotonic decreasing dose-response relationship between LE8 score and predicted probability of MetS (P-overall <0.001; P-nonlinear <0.001).

These results are in line with previous studies demonstrating the utility of ideal cardiovascular health metrics for MetS prediction. The Atherosclerosis Risk in Communities study reported substantially lower prevalence of MetS with better Life’s Simple 7 scores, a precursor of the LE8 metrics [5]. Adopting a healthy lifestyle could substantially reduce premature mortality and prolong life expectancy in US adult [13]. Our study provides corroborating evidence in a nationally representative US population using an enhanced composite measure of cardiovascular health. The dose-response modeling further enriches the characterization of the relationship between LE8 and MetS.

We further identified elevated serum albumin, decreased serum uric acid, and reduced neutrophil count to be significantly associated with lower MetS risk. In particular, participants with serum albumin levels in the highest quartile had a 51% lower likelihood of having MetS compared to those in the lowest quartile (OR: 0.49; 95% CI: 0.43–0.55; P < 0.001). This could be indicative of an improved nutritional status, as serum albumin concentration is a marker of protein nutritional status [5]. Moreover, we found a significant mediating effect of serum albumin, uric acid, and neutrophil levels on the association between LE8 score and risk of MetS, explaining 1.43%, 9.38% and 2.94% of the association, respectively. These findings provide insights into the potential biological mechanisms underlying the link between LE8 metrics and MetS.

Moreover, our study revealed a higher proportion of individuals with advanced education in the MetS group. This may be linked to the fact that highly educated individuals often engage in high-stress occupations, and their actual dietary habits and lifestyle choices may not be healthy, particularly due to neglect of nutritious eating amidst busy work and social activities. Furthermore, this demographic may experience greater psychological stress. These factors can lead to negative impacts on metabolic health [14]. Our analysis also showed that the prevalence of MetS was higher among moderate drinkers compared to heavy drinkers. This could be attributed to a pattern among moderate drinkers of consuming alcohol more frequently but in smaller quantities per occasion, which may differentially affect metabolic health [15]. These results suggest that future research should focus on the impact of different drinking patterns on metabolic syndrome and conduct more detailed studies on behavioral and physiological mechanisms.

Our study identified several circulating biomarkers associated with MetS risk, including serum albumin, uric acid and neutrophils, which may provide insights into the potential mechanisms linking LE8 score to MetS. Low serum albumin could be indicative of impaired nutritional status. Hypoalbuminemia has been associated with higher prevalence of MetS in clinical populations [16]. Albumin is an important carrier protein that maintains colloid osmotic pressure. Nutritional deficiency can reduce albumin synthesis in the liver, compromising its levels and functions [17]. The observed association between lower serum albumin and higher MetS risk may be mediated through malnutrition, suggesting nutrition improvement as a potential avenue to mitigate MetS. Furthermore, lower albumin levels may reflect endothelial dysfunction, a key factor in both cardiovascular health and MetS development, thus potentially explaining part of the relationship between LE8 score and MetS risk.

Elevated serum uric acid has been associated with insulin resistance, obesity and diabetes [18, 19], which are key components of MetS. Uric acid can also promote endothelial dysfunction and vascular smooth muscle cell proliferation, directly impacting cardiovascular health [20]. This dual role in both metabolic and cardiovascular pathways makes uric acid a crucial mediator between LE8 score and MetS risk. The pro-oxidant effects of uric acid at high levels may further exacerbate the oxidative stress associated with both poor cardiovascular health and MetS [21]. Serum uric acid correlates with systemic inflammation [22], and has been linked to defective insulin signaling and pancreatic beta cell dysfunction [23, 24]. The connection between high uric acid and increased MetS risk may be attributable to the interaction between inflammation, altered glucose metabolism and impaired insulin secretion.

Neutrophils play a critical role in the initiation and progression of atherosclerosis, a key component of poor cardiovascular health [25]. Increased neutrophil counts may be indicative of chronic low-grade inflammation, which has been closely linked to MetS pathogenesis [26, 27]. Activated neutrophils can release inflammatory factors, reactive oxygen species and proteases, causing systemic inflammation and tissue damage [28, 29]. The association between high neutrophil count and MetS risk may reflect an underlying pro-inflammatory state, which can promote MetS development and progression to cardiovascular complications.

The LE8 Score incorporates healthy lifestyle factors such as a balanced diet, regular physical activity, adequate sleep, and avoiding smoking and excessive alcohol consumption. These factors can reduce inflammation and oxidative stress, improving insulin receptor signaling and increasing insulin sensitivity [30]. Additionally, a higher LE8 Score may enhance β-cell function by decreasing visceral fat and improving gut microbiota diversity [31]. Insulin resistance and impaired insulin secretion are key mechanisms in the development of hyperglycemia and diabetes [32]. Insulin resistance reduces tissue responsiveness, while secretory defects impair pancreatic β-cell function, both contributing to disease progression [33]. Future research should explore how the LE8 Score mitigates these mechanisms to prevent metabolic syndrome and diabetes.

However, we acknowledge that this study employed a cross-sectional design, which cannot establish causality. Therefore, whether the observed decrease in serum albumin levels, increase in uric acid, and rise in neutrophil count directly cause MetS, or whether these changes are consequences of MetS, remains a subject for further investigation. It is crucial to emphasize that despite our study demonstrating associations between these biomarkers and MetS, the limitations inherent in a cross-sectional design prevent us from excluding the possibility of reverse causality. This means that although we observed that serum albumin, uric acid, and neutrophil count might be associated with MetS through certain mechanisms discussed, these changes could also merely be phenomena that occur following the onset of MetS, rather than its precursors. Thus, caution must be exercised when discussing these biomarkers as potential targets for MetS prevention. Future research should consider employing a longitudinal design, with long-term follow-ups to further verify the causal relationships of these biomarkers in the development of MetS. Additionally, mechanistic studies and intervention trials are also crucial to deepen our understanding of the specific roles these biomarkers play in metabolic health.

Our study has several limitations. First, the cross-sectional design limits causal inference on the relationship between LE8 score and MetS risk. Prospective cohort studies are warranted to establish temporal sequence. Second, the use of self-reported data for estimating some LE8 components may introduce recall bias. Third, we did not evaluate genetic, epigenetic and environmental factors that may interact with LE8 metrics. Finally, our study population from a national sample may limit generalizability to other populations.

Despite these limitations, our study provides robust evidence on the utility of the LE8 score for MetS risk assessment in the general US population. Our results support the value of LE8 score as a population-level screening tool to identify high-risk groups for MetS prevention and treatment. The findings may guide public health policymaking regarding metabolic disease control. Further research is needed to validate the predictive performance of LE8 score across diverse cohorts and settings. Cost-effectiveness analysis of LE8-based screening programs can determine feasibility for implementation in clinical practice. Overall, promoting ideal cardiovascular health behaviors and factors measured in the LE8 score may contribute to alleviating the burden of metabolic disorders in the US. We utilized the NCEP ATPIII as the diagnostic criteria for MetS. These criteria offer straightforward and clear diagnostic indicators, facilitating their practical application by clinicians and researchers. Furthermore, due to its early publication and widespread usage, adopting this standard allows our research findings to be more easily compared and integrated with other studies. In addition, this criterion considers a variety of metabolic risk factors (such as waist circumference, triglycerides, HDL cholesterol, blood pressure, and fasting glucose), providing a comprehensive assessment of the presence and severity of metabolic syndrome. However, a limitation of this standard is the lack of consideration for racial and gender differences [34]. Future studies could employ more comprehensive criteria tailored to specific populations for a thorough evaluation of MetS.

Our findings have raised several promising directions for future research. First, longitudinal studies with long-term follow-up are warranted to verify the causal link between LE8 score and MetS risk. These prospective cohort studies should follow participants over time to explore how changes in LE8 scores affect the incidence of MetS. Such research would help determine the temporal sequence between LE8 score and MetS development, providing stronger evidence for the potential predictive value of the LE8 score in MetS risk assessment. Second, exploring the effects of sociodemographic, lifestyle and genetic factors on the LE8-MetS relationship can elucidate potential modifiers and high-risk subgroups. Third, validating the utility of LE8 metrics in other ethnic populations can support its broader application for MetS screening and prediction. Fourth, further characterizing the dose-response patterns using mediation analysis and machine learning approaches may improve MetS risk stratification based on LE8 score. Fifth, developing and evaluating LE8-based lifestyle interventions for MetS prevention in clinical and community settings is an important next step. Future studies should investigate the role of environmental pollutants in metabolic syndrome risk, focusing on their interaction with LE8 scores. This research should encompass air pollution and persistent organic pollutants, while also examining environmental risk disparities across socioeconomic groups. Such analyses could elucidate the complex relationships between lifestyle, environment, and metabolic health, potentially guiding targeted interventions to reduce metabolic syndrome risk in diverse populations. Finally, cost-effectiveness analyses should determine the feasibility of incorporating LE8 measurement into MetS screening programs. Addressing these future directions can maximize the utility of LE8 metrics for curbing the expanding burden of metabolic disorders worldwide.

## Conclusion

In conclusion, our study based on a large nationally representative sample demonstrates a significant inverse association between LE8 score and risk of metabolic syndrome in US adults. We revealed a monotonic decreasing dose-response relationship between LE8 metrics and predicted probability of MetS. Our results support the value of promoting ideal cardiovascular health behaviors and factors encompassed in the LE8 metric to curb the rising burden of metabolic disorders. Further prospective studies validating the predictive performance and clinical utility of LE8 score are warranted.

## Supporting information

S1 Table Definition and scoring approach for the American Heart Association’s Life Essentials 8 score. (DOCX)

S2 Table Healthy Eating Index-2015 components & scoring standards. (DOCX)
