# Supplementary material for: Life Essentials 8 score and risk of metabolic syndrome: A dose-response analysis in the US population
Source: PLoS One. 2024 Oct 31;19(10):e0312674. doi: 10.1371/journal.pone.0312674 (PMC11527257; doi:10.1371/journal.pone.0312674)
Supplement: S1 Table — (DOCX) [file pone.0312674.s001.docx]

S1 Table. Definition and scoring approach for the American Heart Association’s Life’s Essential 8 score

| Domain | CVH Metric | Measurement | Quantification and Scoring of CVH Metric |
| --- | --- | --- | --- |
| Health Behaviors | Diet | Healthy Eating Index-2015 diet score percentile | Quantiles of DASH-style diet adherence  **Scoring (Population):**  Points Quantile  100 ≥95^th^ percentile (top/ideal diet)  80 75^th^ – 94^th^ percentile  50 50^th^ – 74^th^ percentile  25 25^th^ – 49^th^ percentile  0 1^st^ – 24^th^ percentile (bottom/least ideal quartile) |
|  | Physical activity | Self-reported minutes of moderate or vigorous physical activity per week | **Metric:** Minutes of moderate (or greater) intensity activity per week  **Scoring:**  Points Minutes  100 ≥150  90 120 – 149  80 90 – 119  60 60 – 89  40 30 – 59  20 1 – 29  0 0 |
|  | Nicotine exposure | Self-reported use of cigarettes or inhaled nicotine- delivery system | **Metric:** Combustible tobacco use and/or inhaled NDS use; or secondhand smoke exposure  **Scoring:**  Points Status  100 Never smoker  75 Former smoker, quit ≥5 yrs  50 Former smoker, quit 1 - <5 yrs  25 Former smoker, quit <1 year, or currently using inhaled NDS  0 Current smoker  Subtract 20 points (unless score is 0) for living with active indoor smoker in home |
|  | Sleep health | Self-reported average hours of sleep per night | **Metric:** Average hours of sleep per night  **Scoring:**  Points Level  100 7 – <9  90 9 – <10  70 6 – <7  40 5 – <6 or ≥10  20 4 – <5  0 <4 |
| Health Factors | Body mass index | Body weight (kg) divided by height squared (m^2^) | **Metric:** Body mass index (kg/m^2^)  **Scoring:** Points Level 100 <25  70 25.0 – 29.9  30 30.0 – 34.9  15 35.0 – 39.9  0 ≥40.0 |
|  | Blood lipids | Plasma total and HDL-cholesterol with calculation of non-HDL-cholesterol | **Metric:** Non-HDL-cholesterol (mg/dL)  **Scoring:**  Points Level  100 <130  60 130 – 159  40 160 – 189  20 190 – 219  0 ≥220  If drug-treated level, subtract 20 points |
|  | Blood glucose | Fasting blood glucose or casual hemoglobin A1c | **Metric:** Fasting blood glucose (mg/dL) or Hemoglobin A1c (%)  **Scoring:**  Points Level  100 No history of diabetes and FBG <100 (or HbA1c < 5.7)  60 No diabetes and FBG 100 – 125 (or HbA1c 5.7-6.4) (Pre-diabetes)  40 Diabetes with HbA1c <7.0  30 Diabetes with HbA1c 7.0 – 7.9  20 Diabetes with HbA1c 8.0 – 8.9  10 Diabetes with Hb A1c 9.0 – 9.9  0 Diabetes with HbA1c ≥10.0 |
|  | Blood pressure | Appropriately measured systolic and diastolic blood pressure | **Metric:** Systolic and diastolic blood pressure (mm Hg)  **Scoring:**  Points Level  100 <120/<80 (Optimal)  75 120-129/<80 (Elevated)  50 130-139 or 80-89 (Stage I HTN)  25 140-159 or 90-99  0 ≥160 or ≥100  Subtract 20 points if treated level |

**Reference**

1. Lloyd-Jones DM, Allen NB, Anderson CAM, et al. Life's Essential 8: Updating and Enhancing the American Heart Association's Construct of Cardiovascular Health: A Presidential Advisory From the American Heart Association. *Circulation*. Aug 2 2022;146(5):e18-e43.
2. Lloyd-Jones DM, Ning H, Labarthe D, et al. Status of Cardiovascular Health in US Adults and Children Using the American Heart Association's New "Life's Essential 8" Metrics: Prevalence Estimates From the National Health and Nutrition Examination Survey (NHANES), 2013 Through 2018. *Circulation*. Sep 13 2022;146(11):822-835.
